# Drug-induced liver injury following the use of tocilizumab or sarilumab in patients with coronavirus disease 2019

**DOI:** 10.1186/s12879-022-07896-0

**Published:** 2022-12-12

**Authors:** Qian Gao, Xuedong Yin, Boyu Tan, Junshi Wang, Jiayan Chen, Bin Zhao, Qiaoling Yang, Zhiling Li

**Affiliations:** 1 Department of Pharmacy, Shanghai Children’s Hospital, School of Medicine, Shanghai Jiao Tong University, Shanghai, 200062 China; 2 School of Medicine, Shanghai Jiao Tong University, Shanghai, 200125 China; 3 Macau University of Science and Technology, Macau, China; 4 School of Nursing and Health, Shanghai Zhongqiao Vocational and Technical University, Shanghai, 201514 China; 5 Department of Pharmacy, Peking Union Medical College Hospital, Peking Union Medical College, Chinese Academy of Medical Sciences, Beijing, 100730 China; 6 Institute of Chinese Materia Medica, Shanghai University of Traditional Chinese Medicine, Shanghai, 201203 China

**Keywords:** Coronavirus disease 2019 (COVID-19), Tocilizumab, Sarilumab, Drug Induced Liver Injury, FAERS, Epidemiology

## Abstract

**Backgrounds:**

Interleukin-6 (IL-6) blockers including tocilizumab and sarilumab were approved by the U.S. Food and Drug Administration (FDA) in June 2021 for the treatment of patients with moderate to severe COVID-19. The use of sarilumab or tocilizumab in COVID-19 patients has been related to a reduction in mortality compared to standard care. Recent evidence has emerged concerning drug-induced liver injury (DILI) after sarilumab or tocilizumab applications in COVID-19 patients.

**Aims:**

The study aimed to estimate DILI associated with sarilumab or tocilizumab in treating moderate to severe patients infected with SARS-Cov-2.

**Methods:**

We conducted a retrospective pharmacovigilance study by data mining of the FDA’s adverse event reporting systems (FAERS) database from the first quarter of 2004 to the fourth quarter of 2021 in confirmed COVID-19 patients. We analyzed DILI cases associated with tocilizumab or sarilumab in treating COVID-19 patients from the FAERS during this period. Disproportionality analysis and Bayesian analysis of COVID-19 patients were utilized for case analysis, and we also next compared the onset time and fatality rates of DILI following tocilizumab or sarilumab.

**Results:**

A total of 275 cases of TCZ or SAR-related DILI reports were extracted. A total of 192 AEs cases were related to tocilizumab (TCZ), and 83 were related to sarilumab (SAR). In patients treated with TCZ, most were < 75 years old (51.57%), with more male than female (46.35% *vs*. 13.02%). The correlation between IL-6 receptor antagonists and DILI was stronger in SAR (ROR = 12.94; 95%CI 9.6–17.44) than in TCZ (ROR = 1.33; 95%CI 1.14–1.55). The onset time of DILI was different between TCZ and SAR, and a significant difference was observed in TCZ than SAR (*P* < 0.0001). A significant difference was observed in the mortality rate of TCZ and SAR (*P* = 0.0009). DILI associated with COVID-19 patients treated with TCZ appeared to have earlier onset-time (1(0–46) day) VS. SAR (3.5(0–27) day).

**Conclusion:**

This study shows strict monitor ought to be paid for TCZ or SAR when used for COVID-19 patients with poor liver function.

**Supplementary Information:**

The online version contains supplementary material available at 10.1186/s12879-022-07896-0.

## Introduction

At the end of 2019, the severe acute respiratory syndrome coronavirus 2 (SARS-CoV-2) emerged as a new human pathogen. This unknown virus, which causes coronavirus disease 2019 (COVID-19), was named by the WHO on February 11, 2020. On March 11, 2020, the WHO declared COVID-19 a global pandemic causing enormous economic and social disruption worldwide. As of March 20, 2022, there were more than 468 million confirmed cases of COVID-19 worldwide, including approximately 6 million deaths [1]. Severe cytokine release syndrome (CRS) is a life-threatening acute systemic inflammatory response characterized by multiple organ failure and fever. It plays a central role in the pathogenesis of SARS-CoV-2 infection. SARS-CoV-2 infection induces dose- and time-dependent production of cytokines. In severe COVID-19 disease, elevated levels of cytokines, especially interleukin 6 (IL-6), have been observed and were considered a critical factor in inflammation [2, 3]. COVID-19 patients with elevated IL-6 levels and evidence of hyperinflammation had increased rates of more severe symptoms [4, 5]. These clinical findings can provide new ideas for the treatment of COVID-19.

Various options have been used to treat COVID-19, such as remdesivir, lopinavir/ritonavir, umifenovir, convalescent plasma, SAR, TCZ, inhalational budesonide, methylprednisolone, hydroxychloroquine, azithromycin, and mesenchymal stem cells [6]. IL-6 blockers can significantly reduce the risk of death and mechanical ventilation in moderate to severe patients compared to other treatments while also causing fewer serious adverse effects [7, 8]. IL-6 blockers are classified as anti-IL-6 receptor monoclonal antibodies (SAR, TCZ) (Table 1) or anti-IL-6 monoclonal antibodies (siltuximab, olokizumab). Some studies show a potentially clinically significant impact of TCZ on lung function preservation [9, 10]. TCZ (molecular weight is 148KDa) and SAR (molecular weight is 150KDa) are humanized monoclonal antibodies that bind to membrane-bound and soluble IL-6 receptors to inhibit the IL-6 signalling pathway by blocking dimerization of glycoprotein (gp)130 molecules on the cell membrane [11–13]. In a population pharmacokinetic study observing 1793 RA patients, the patients received a 1-h infusion of TCZ 4 or 8 mg/kg every 4 weeks for 24 weeks. In the population pharmacokinetic (PK) analysis, a dose proportional increas in maximum concentration (Cmax) was observed after TCZ 4 or 8 mg/kg every 4 weeks, however, the increasing in the area under the concentration–time (AUC) curve and the through concentration was more than dose proportional. Ohsugi et al. found that in vivo, sIL-6R saturation maximum (> 90%) when serum TCZ concentrations were > 1ug/ml [14, 15]. Compared with TCZ, the binding kinetics and functional activity of SAR were more higher and SAR bound to IL-6R with higher affinity than TCZ [16]. In a PK characterization of SAR in patients with RA including subcutaneous SAR 150 mg or 200 mg every 2 weeks for up to one year, a greater than dose-proportional increase in SAR exposure was observed [14].

**Table 1:** Summary of FDA-approved IL-6 receptor antagonist

Generic name	Brand name	Indication	Year of approval by FDA
Tocilizumab	Actemra	RA, CRS, sJIA, Giant cell arteritis, COVID-19	2003; tocilizumab was granted an EUA for the treatment of COVID-19 in June 2021
Sarilumab	Kevzara	RA	2017

*RA* rheumatoid arthritis, *CRS* cytokine release syndrome, *sJIA* systemic juvenile idiopathic arthritis

IL-6 receptor antagonists are used to treat some hyperinflammatory diseases, such as rheumatoid arthritis [17], giant cell arteritis [18], and cytokine release syndrome (CRS) induced by chimeric antigen receptor (CAR) T-cell therapy [19]. COVID-19 can attack the immune system, causing it to over-react and produce dangerous levels of inflammatory factors [20]. Therefore, IL-6 receptor antagonists have been used to treat severe COVID-19. In a randomized clinical trial (RCT) conducted in England involving 452 hospitalized patients with severe COVID-19, the use of TCZ did not result in a significantly better prognosis or lower mortality than placebo at 28 days (19.7% in the TCZ and 19.4% in the placebo groups) [21]. In another RCT involving 389 patients with COVID-19 who were not receiving mechanical ventilation, TCZ reduced the probability of progression of mechanical ventilation or death compared to the placebo group (12.0% in the TCZ and 19.3% in the placebo groups) [22].

Adverse events (AEs) of TCZ in treating non-COVID diseases are upper respiratory tract infection, urinary tract infection, nasopharyngitis, hypercholesterolemia, and neutropenia. Hypertension and gastrointestinal perforation are also reported [23]. In patients with severe or critical COVID-19, AEs were reported in 43% of patients who received TCZ compared to 34% who did not (P = 0.26) [24]. A phase III clinical trial in India showed that 36% of patients in the TCZ group and 25% of patients in the standard care group had AEs. Serious AEs occurred 20% in the TCZ group and 17% in the standard care group. In this study, involving 180 patients, 33 of 91(36%) patients in the TCZ group and 22 0f 89(25%) patients in the standard care group had AEs; 18 of 91(20%) and 15 of 89(17%) had serious AEs; 13 of 91(14%) and 15 of 89(17%) patients died during the study [25].

Currently, there is no systematic retrospective analysis that summarizes the AEs of IL-6 receptor antagonists in treating patients with COVID-19. Our study aimed to use the US Food and Drug Administration Adverse Event Reporting System (FAERS) to analyse and compare the differences and links of DILI in COVID-19 patients receiving TCZ or SAR to supply a reference for clinicians to treat COVID-19. We further examined the onset-time and fatality and hospitalization rates for DILI of COVID-19 patients treated with TCZ or SAR.

## Method

### Data source

The FAERS database, a publicly available spontaneous reporting system, is typically used for initial investigations of new or rare AEs and for evaluating drug-drug interactions. The database contains detailed information on the demographics of medications, indications, outcomes, and sources of reports. All AEs were coded using Preferred Terms (PTs) in the Medical Dictionary for Regulatory Activities (MedDRA, version 25.0) Terminology. The MedDRA hierarchy contains five levels, system organ class (SOC), high level group term (HLGT), high level term (HLT), preferred term (PT), lowest level term (LLT). The “System Organ Classes,” grouped by disease etiology, manifestation site, or purpose, are the high-tier group terms. PTs, which describe disease symptoms or diagnosis, indications, laboratory results, and procedures, are low-level group terms used in the FAERS database [6].

**Fig. 1:**
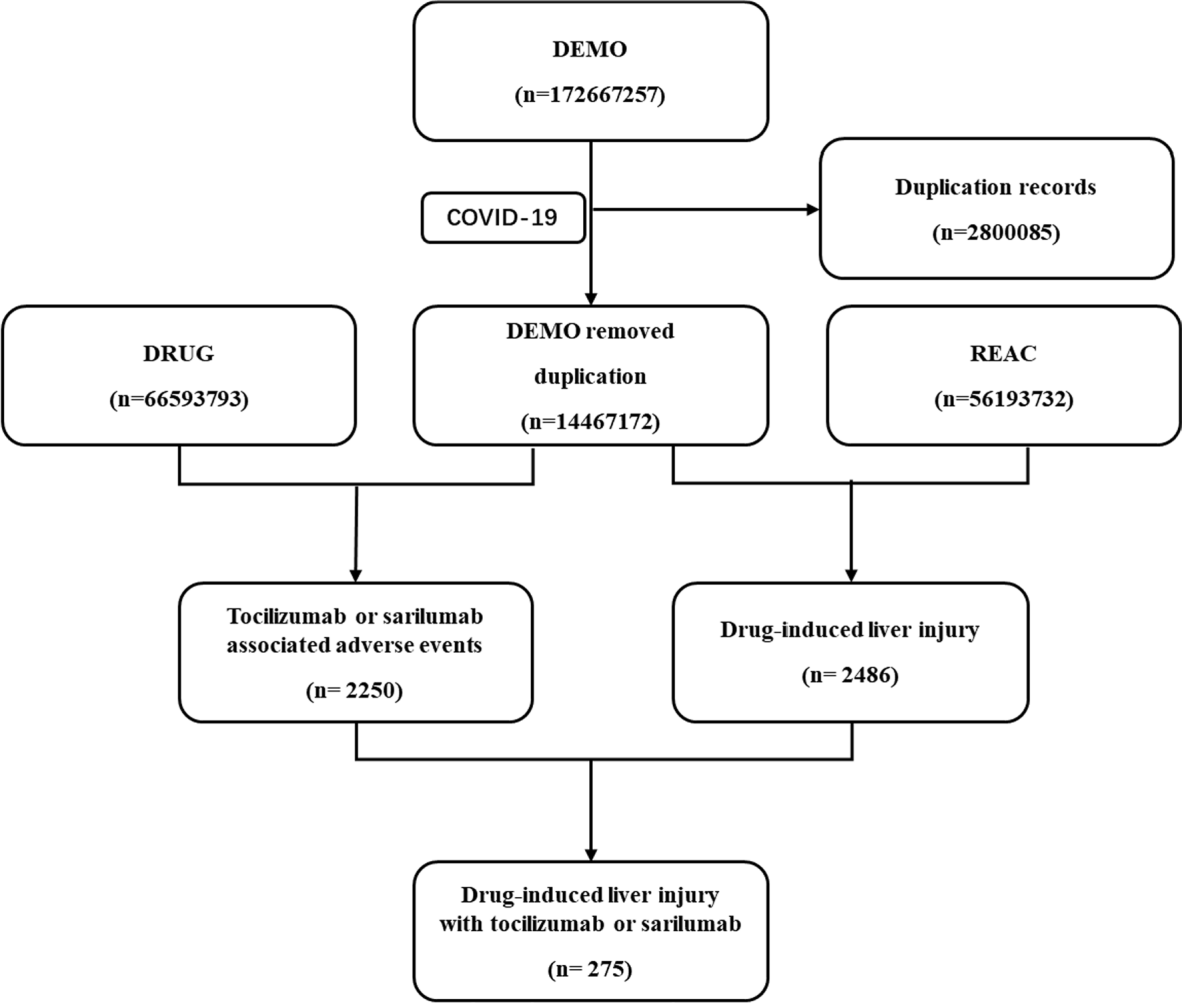
Process of the selection of cases of IL-6 receptor antagonists-associated liver injury from the FAERS database. *DEMO* demographic information, *DRUG* drug information, *REAC* adverse events

Data were extracted from the FAERS database from the first quarter of 2004 to the fourth quarter of 2021 and we obtained a total of 17,267,257 reports from the FEARS database (Fig. 1). From these data, we extracted COVID-19 cases from the second quarter of 2020 to the fourth quarter of 2021. We removed the duplicate records according to FDA’ recommendations, and we finally identify 192 cases of DILI associating with TCZ as a suspected drug, and 83 cases by SAR.

### Identification of coronavirus infection

We searched the case reports of COVID-19 patients with DILI after treatment with TCZ or SAR, from the second quarter of 2020 to the fourth quarter of 2021. During the search, we use keywords as: COVID-19, 2019-Ncov, SARS-Cov-2.

### Drug identification and adverse drug event classification

Drug-induced liver injury is based on the Preferred Term of the MedDRA, and we searched the FAERS database to confirm COVID-19 patients cases treated with TCZ (also called actemra, atlizumab, roactemra) and SAR (also called Kevzara). We focus on adverse effects in COVID-19 patients treated with TCZ or SAR. In this study, we examined TCZ and SAR according to the COVID-19 treatment guidelines and considered DILI was associated with these two drugs. The MedDRA preferred terms used to interpret hepatotoxicity cases of TCZ and SAR are shown in Table 2.

**Table 2:** MedDRA preferred terms that used to retrieve adverse event cases of IL-6 receptor antagonists in FAERS

PT (preferred terms)
LIVER INJURY
LIVER DAMAGE
HEPATIC DAMAGE
HEPATOTOXICITY
HEPATITIS
NONALCOHOLIC FATTY LIVER DISEASE
LIVER FATTY INFILTRATION
CHOLESTASIS
BILIARY CHOLANGITIS
HEPATIC ENCEPHALOPATHY
HEPATIC FAILURE
PORTAL HYPERTENSION
HEPATIC NECROSIS
DILI
HEPATOCELLULAR INJURY
HEPATIC ENZYME ABNORMAL
BLOOD BILIRUBIN ABNORMAL

### Data mining and statistical analysis

Data mining algorithms detect FAERS signals, where a signal means a statistical association between drugs and drug-associated adverse events [11]. These algorithms consist of the proportional reporting ratio (PRR), the reporting odds ratio (ROR), the information component (IC), and the empirical Bayes geometric mean (EBGM) [26]. PRR and ROR are non-Bayesian disproportionality analyses, whereas the IC and EBGM are Bayesian analyses. A two-by-two contingency table was constructed to identify signals of reported event counts for TCZ and SAR (Table 3).

**Table 3:** A two-by-two contingency table for a drug-adverse event combination

	With an adverse event of interest	Other adverse events	Total
With a drug of interest	a	b	(a + b)
Without a drug of interest	c	d	(c + d)
Total	(a + c)	(b + d)	(a + b + c + d)

PRR = (a/[a + b])/(c/[c + d]) (criteria: PRR ≥ 2,χ2 ≥ 4, n ≥ 3); ROR = (a/c)/(b/d), 95% CI = eln^(ROR) – 1.96(1/a+1/b+1/c+1/d)^0.5^ (criteria: 95% CI > 1, n ≥ 2); IC = log2a*(a + b + c + d)/([a + b][a + c]), IC025 = e^ln(IC)–1.96(1/a+1/b+1/c+1/d)^0.5^ (criteria:IC025 > 0); EBGM = a*(a + b + c + d)/([a + b][a + c]), EBGM05 = e^ln(EBGM)–1.64(1/a+1/b+1/c+1/d)^0.5^ (criteria: EBGM05 > 2, n > 0). *CI* confidence interval, *n* indicates the number of co-occurrences, *χ2* chi-squared, *IC* information component, *IC025* the lower limit of the 95% two-sided CI of the IC, *EBGM05* the lower limit of the 90% one-sided CI of the EBGM

We also estimated the onset time of DILI with TCZ or SAR in COVID-19 patients, defined as time interval between the onset-time of DILI and the date of initiation of the medication, and we removed entered erroneously (Reports with EVENT_DT earlier than START_DT) or inaccurate date entries. In addition, we analysed the reports of fatal events in COVID-19 patients due to DILI, which were calculated by dividing the number of fatal events by the total number of TCZ- or SAR-associated DILI events.

GraphPad Prism 8.0.2(GraphPad Software, CA, USA) was used to statistically analyse the DILI of COVID-19 patients treated with TCZ or SAR. The two-tailed Student's t-test was used to analyse between-group analysis differences, and the difference between multiple groups was investigated by the Kruskal–Wallis test. P < 0.05 was considered statistically significant.

## Results

### Descriptive analysis

From May 2020 to December 2021, the FAERS database had 2486 adverse events reports in COVID-19 patients treated with monoclonal antibody. Among these cases, we filtered out 192 cases with suspected TCZ-associated DILI and 83 cases with suspected SAR-associated DILI. From the analysis results, we found that the IL-6 receptor antagonists used for the treatment of COVID-19 were mainly TCZ and SAR. Table 4 summarizes the clinical characteristics of these patients. These cases were concentrated in 2021. 80.73% of the adverse reactions related to DILI reported by TCZ for COVID-19 treatment were from patients in North America and Europe, while 91.56% of SAR were from North America and Europe. For the patient age, eliminating unknown data, adverse reactions associating with DILI are mainly concentrated between the ages of 45–64, respectively TCZ 30.21% and SAR 3.61%. Surprisingly, 96.39% of cases on SAR-related DILI were reported without mentioning age. Further, the AEs of these two monoclonal antibodies were more in females than in males (TCZ vs. SAR, 46.35% vs. 2.41%), excluding unknown data, meanwhile, 96.39% of cases on SAR-related DILI were reported without regard to gender. Additionally, SAR-related DILI cases were mainly uploaded by physicians (98.8%), and TCZ-related cases were uploaded by pharmacists (27.08%) and physicians (33.33%). And no SAR-related DILI cases have been reported to FAERS database after the first quarter of 2021 (Additional file 1).

**Table 4:** Clinical characteristics of COVID-19 patients with IL-6 receptor antagonists-associated DILI sourced from the FAERS database (2020q2 to 2021q4)

Characteristics	Drug report, n (%)	
	TCZ	SAR
*Patient age (year)*		
18–44	20 (10.42%)	0 (0.00%)
45–64	58 (30.21%)	3 (3.61%)
65–74	21 (10.94%)	0 (0.00%)
75–84	11 (5.73%)	0 (0.00%)
≥ 85	2 (1.04%)	0 (0.00%)
Unknown	80 (41.67%)	80 (96.39%)
*Patient gender*		
Female	25 (13.02%)	1 (1.20%)
Male	89 (46.35%)	2 (2.41%)
Unknown	78 (40.63%)	80 (96.39%)
*Reporter occupation*		
Consumer	2 (1.04%)	0 (0.00%)
Pharmacist	52 (27.08%)	1 (1.20%)
Physician	64 (33.33%)	82 (98.80%)
Unknown	74 (38.54%)	0 (0.00%)
*Report time*		
2020Q2	13 (6.77%)	6 (7.23%)
2020Q3	29 (15.10%)	13 (15.66%)
2020Q4	23 (11.98%)	61 (73.49%)
2021Q1	20 (10.42%)	3 (3.61%)
2021Q2	53 (27.60%)	0 (0.00%)
2021Q3	25 (13.02%)	0 (0.00%)
2021Q4	29 (15.10%)	0 (0.00%)
*Area*		
Africa	2 (1.04%)	0 (0.00%)
Asian	22 (11.46%)	0 (0.00%)
Europe	102 (53.13%)	13 (15.66%)
North America	53 (27.60%)	63 (75.90%)
South America	13 (6.77%)	7 (8.43%)
*Outcomes*		
Congenital anomaly	0 (0.00%)	1 (1.22%)
Death	30 (17.14%)	30 (36.59%)
Disability	1 (0.57%)	1 (1.22%)
Hospitalization initial or prolonged	27 (15.43%)	37 (45.12%)
Life-threatening	6 (3.42%)	29 (35.37%)
Other serious (important medical event)	137 (78.29%)	54 (65.85%)
Required intervention to prevent permanent impairment	2 (1.14%)	0 (0.00%)

### Disproportionality analysis and Bayesian analysis

The data showed a strong association between TCZ, SAR, and DILI. Intraclass analysis of the correlation between IL-6 receptor antagonist and DILI showed the following ranking: SAR (ROR = 12.94; 95%CI 9.6–17.44) > TCZ (ROR = 1.33; 95%CI 1.14–1.55) (Table 5). Notably, SAR has more strong association with DILI than TCZ because of its stronger signal.

**Table 5:** The association of TCZ and SAR with DILI

Drugs	N	ROR	PRR	IC	EBGM
		(95% two-sided CI)	(χ2)	IC025	EBGM05
TCZ	191	1.33 (1.14, 1.55)	1.3 (13.27)	0.36 (0.31)	1.28 (1.13)
SAR	83	12.94 (9.6, 17.44)^*^	7.31 (468.91)^*^	2.83 (2.1)^*^	7.11 (5.54)^*^

TCZ-associated DILI ROR=1.33, 95% two-sided CI(1.14,1.55), PRR=1.3, χ2=13.27, EBGM=1.28, 95% one-sided CI=1.2; SAR-associated DILI ROR=12.94, 95% two-sided CI(9.6,17.44), PRR=7.31, χ2=468.91, EBGM=7.11, 95% one-sided CI=5.54; * means a strong signal that represents a strong statistical association between the drug and the adverse reaction

### Onset times of IL-6 receptor antagonists-associated DILI

**Fig. 2:**
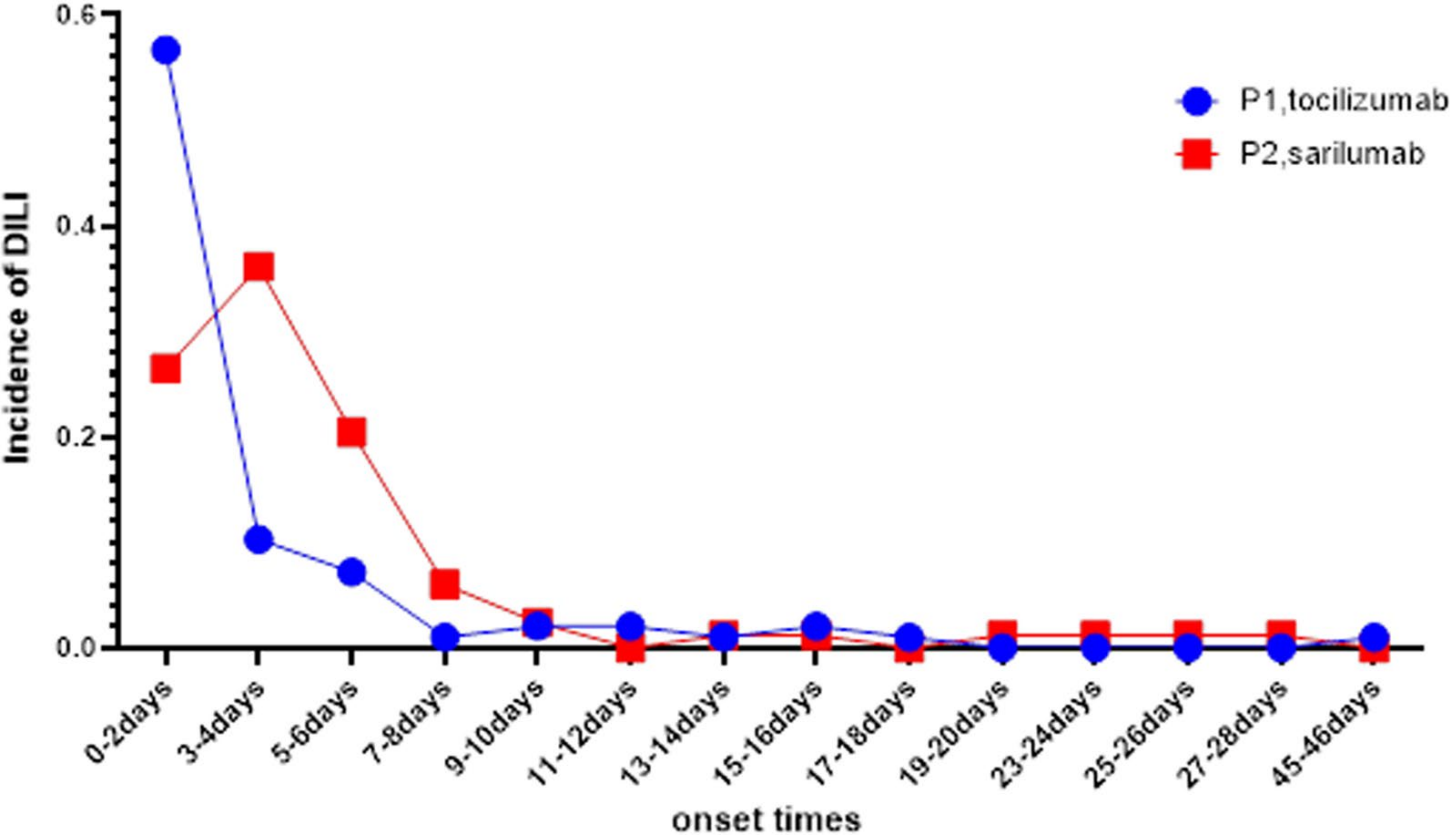
The difference in the peak onset times of adverse reactions between TCZ and SAR

The median onset time of the DILI for TCZ is 1(IQR 0–46) day and SAR is 3.5(IQR 0–27) days. The onset time of DILI was different between these two IL-6 inhibitors. Significant differences were observed for TCZ versus SAR (two-tailed Student's t-test, P < 0.05). There is also a noticeable difference in the peak onset times of adverse reactions between the two drugs (Fig. 2). According to the data, TCZ-related DILI accounted for 67.01% the first four days after receiving TCZ therapies, and SAR-associated DILI accounted for 83.13% the first six day after receiving SAR therapies. Notably, we found that DILI may occur after the first dose of TCZ in treating COVID-19, whereas SAR showed a peak after the last dose, which may due to different compositions and structures result to different pharmacokinetic (PK) and pharmacodynamic (PD).

### Outcomes of TCZ and SAR

**Fig. 3:**
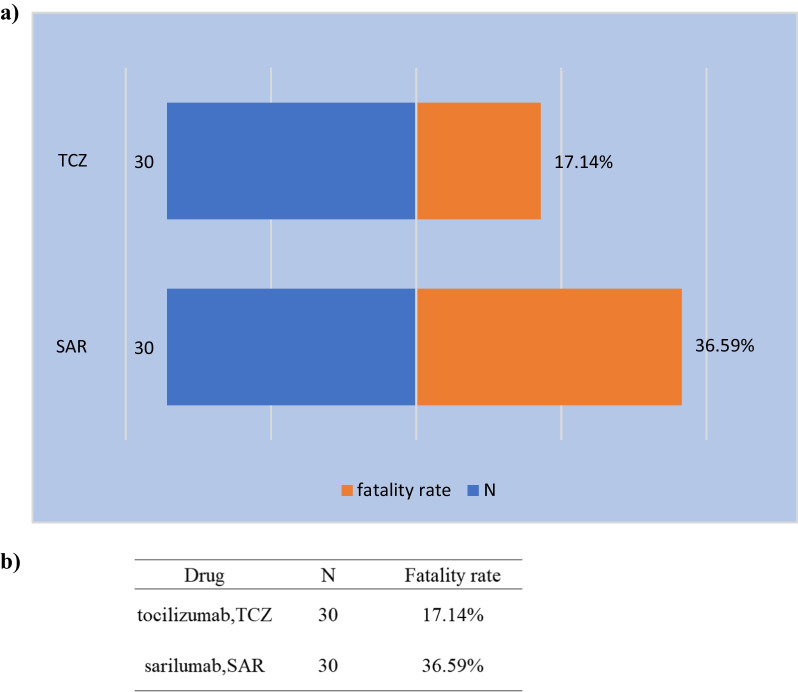
The number of reports and fatality rate for DILI following TCZ or SAR therapy

To analyse the prognosis of patients with COVID-19 treated with IL-6 receptor antagonists, we calculated the proportions of outcomes (death, disability, initial or prolonged hospitalization, life-threatening, and other serious conditions requiring interventions) after receiving TCZ or SAR therapies, and the results are shown in Table 6. Among COVID-19 patients treated with TCZ,17.14% of patients died, and 15.4% were hospitalized compared with SAR, 36.6% died, 45% hospitalized. Additionally, there was a significant difference in the mortality rate of TCZ and SAR (*P* = 0.0009). The mortality rates for these two drugs are shown in Fig. 3. Despite the significant difference in death proportions, we could not be certain that receiving TCZ or SAR was associated with mortality in COVID-patients, the reason is that, in the FEARS database, the sample capacity of COVID-19 patients treated with TCZ and SAR is not consistent.

**Table 6:** The outcomes of TCZ and SAR

Outcome	TCZ (N, %)	SAR (N, %)
Congenital anomaly	0 (0.00)	1 (0.01)
Death	30 (0.17)	30 (0.37)
Disability	1 (0.01)	1 (0.01)
Hospitalization-initial or prolonged	27 (0.15)	37 (0.45)
Life-threatening	6 (0.03)	29 (0.35)
Other serious/important medical events	137 (0.78)	54 (0.66)
Required intervention to prevent permanent impairment/damage	2 (0.01)	0 (0.00)

## Discussion

Infection by SARS-CoV-2 induces a dose-dependent production of IL-6 from bronchial epithelial cells [27]. Elevated IL-6 levels are associated with poor prognosis in COVID-19, including respiratory failure and death. Furthermore, IL-6 plays a critical role, not only in metabolic function but, in the regulation of multiple physiological systems, including tissue homeostasis such as liver regeneration [28]. The liver is an important organ for metabolic activities, and abnormal liver function will be accompanied by an increase in the level of transaminase [29]. IL-6 receptor blockers neutralize membrane-bound and soluble forms of the IL-6 receptor, blocking the activation of cytokines and regulation of the immune response to infection. IL-6 receptor blockers are monoclonal antibodies approved for use in rheumatoid arthritis (RA) by FDA, respectively TCZ in 2013 and SAR 2017. Based on a review of the data from RECOVERY clinical trial (NCT #04381936), the COVACTA clinical trial (NCT #04320615), the EMPACTA clinical trial (NCT#04372186) and the REMDACTA clinical trial (NCT #04409262), FDA believed that TCZ may be effective for the treatment of COVID-19 patients and approved it for the treatment of COVID-19 in June, 2021. WHO strongly recommend treatment with IL-6 receptor antagonist for patients with severe or critical COVID-19 [30]. COVID-19 treatment guidelines recommended SAR may be used as an alternative if TCZ is not available because it has demonstrated a similar clinical benefit in improving survival and reducing the duration of organ support in the REMAP-CAP trial [31].

In this disproportionality analysis of FAERS database, we detected signals of increased risks of DILI associated with TCZ versus SAR in patients with COVID-19. Our work is a collection until recently to compare the associations, onset-time, prognosis of COVID-19 patients with DILI after using TCZ or SAR in the real-world based on the FAERS database. In this study, we noticed that TCZ or SAR-related DILI affected more man than woman. Eliminating unknown data, we found that 46.35% of the COVID-19 patients who received TCZ therapy were male and 13.02% were female, which is similar to the gender composition of patients in other RCTs, with more male than female participants [32]. TCZ was initially approved by the FDA for the treatment of RA, which is more common in middle-aged women, but more men than women have AEs among COVID-19 patients treated with TCZ [33, 34]. We may conclude that TCZ treatment of the COVID-19 patients is might be related to more AEs because the drugs population was dominated by female patients, but the AEs after medication were dominated by males. Mao et al. reported that the incidence of liver injury in male patients with COVID-19 is higher than that in female patients [35], but whether it is related to TCZ treatment remains to be further verified. It is a pity that 96.39% of the COVID-19 patients who received SAR therapy were not reported gender in FAERS database, so there is insufficient evidence for analysing the connection between SAR-associated DILI and gender.

We also noticed that among the COVID-19 patients, except for the unknown data, among the patients receiving TCZ treatment, the elderly over 65 years old accounted for 17.71%, and in the SAR, the proportion of the elderly over 65 years old was 0; surprisingly, 96.39% of the COVID-19 patients who received SAR therapy were also not reported age which is similar with gender report. A RECOVERY clinical trial [36] in the United Kingdom, participants’ mean age is 64 years-old. Among the reporters, except for the unknown data, for the COVID-19 patients receiving TCZ treatment, consumers accounted for 1.04%, pharmacists accounted for 27.08%, and physicians accounted for 33.33%; while for the COVID-19 patients receiving SAR treatment, no consumer reported data, and pharmacists accounted for 1.20%, physicians accounted for 98.8%. SAR treated the COVID-19 patients as an alternative if TCZ is not available because the evidence of efficacy for TCZ is more extensive than for SAR. In terms of reporting time, the proportion of SAR reported in Q4 in 2020 is as high as 73.49%; after 2021Q1, the proportion of SAR adverse reactions reported is 0. Compared to SAR, DILI caused by TCZ has the highest reporting rate in 2021Q2, reaching 27.6%, which probably because FDA issued an Emergency Use Authorizations (EUA) for TCZ for the treatment of COVID-19 in hospitalized adults and pediatric patients [37].

**Fig. 4:**
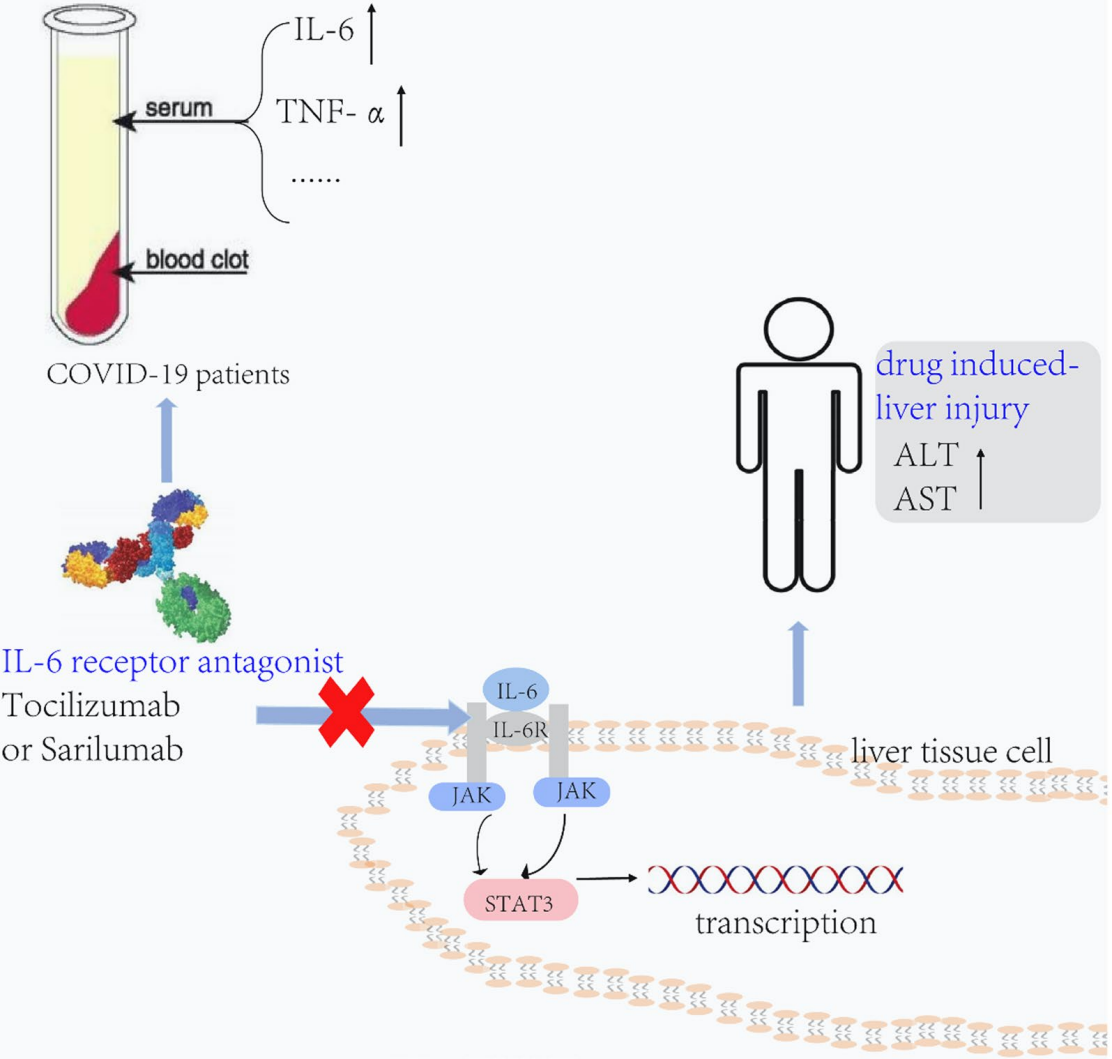
Mechanism of liver injury caused by IL-6 receptor antagonist therapy for COVID-19 patients

Deaths accounted for 17.14% of the 192 cases of DILI associated with TCZ; among the 83 COVID-19 patients treated with SAR, deaths accounted for 36.59%. Many factors can affect the pharmacokinetics of IL-6 inhibitors, such as age, weight, body surface area, comorbidities, and treatment with other drugs. AUC_0-14_ day doubled when the SAR usage dose increased from 150 mg/kg (every two weeks) to 200 mg/kg (every two weeks) [38]. The clearance of TCZ is concentration-dependent, related to body surface area, and its half-life increases with dose and frequency of administration [39]. In this study, we highlighted the effects of COVID-19 patient age and gender on the prognosis of IL-6 receptor antagonist treatment in the FAERS database. We found that SAR (ROR = 12.94; 95% two-sided CI 9.6–17.44) was more strongly associated with DILI than TCZ (ROR = 1.33; 95% two-sided CI1.14–1.55). The results are consistent with a previous systematic review finding that TCZ has a more uncertain association with DILI than SAR [32]. The primary laboratory abnormalities reported with TCZ treatment are elevated liver enzyme levels that appear to be dose-dependent [23]. Muhovic et al. also reported a 52-year-old patient with COVID-19 who was healthy in the past. On the second day after receiving TCZ treatment, ALT increased from normal level to 1541U/L, AST increased from normal level to 1076U/L, and a few days after drug withdrawal, liver function returns, which shows that TCZ treatment can lead to liver damage in COVID-19 patients [40]. According to past reports, the effect of TCZ on liver function may be related to blocking IL-6. IL-6-TAK-STAT3 pathway is a significant signal for liver regeneration, and the binding of IL-6 to its receptor can activate the JAK/STAT3 signalling pathway to promote hepatocyte proliferation [41, 42]. TCZ inhibits the binding of IL-6 to its receptor to block the JAK/STAT3 signalling pathway, which induces severe DILI by promoting hepatocyte apoptosis and inhibiting liver regeneration [43] (Fig. 4).

For pregnant women, there are insufficient data to determine whether there is a TCZ or SAR-associated risk for major birth defects or miscarriage. mAbs are actively transported across the placenta as pregnancy progresses, and this may affect immune responses in the exposed fetus. Given the lacking of data, current recommendations advise against the use of IL-6 receptor antagonists during pregnancy [44]. There are no systematic observational or randomized controlled trial data on the effectiveness of TCZ or SAR for the treatment of acute COVID-19 in pediatric patients. So there is insufficient evidence for the Panel to recommend either for or against the use of TCZ or SAR in hospitalized children with COVID-19.

Data from COVID-19 patients obtained from the FAERS database in this study showed that ARs occurred mostly within two days after TCZ treatment compared to four days after SAR treatment. The onset time difference also reached statistical significance (P = 0.0292). In previous research, IL-6 returned to baseline levels on day 14 after a subcutaneous injection of low-dose SAR and on day 20 after a subcutaneous injection of low-dose TCZ. The peak time for IL-6R after SAR treatment was earlier than for TCZ, ranging from 11–13 days for SAR and 19–22 days for TCZ [45]. In addition, we also found that the proportion of COVID-19 patients who died in ARs reports after SAR therapies were significantly higher than that of TCZ, which may explain why clinicians are more willing to use TCZ than SAR in patients with severe COVID-19.

Our study has the following limitations: (1) the FAERS database has incomplete information such as sex, age, and reporting area, and (2) this is a retrospective study based on spontaneous submitting data, so we could not assess the causality between DILI and TCZ with SAR, and (3) confounding factors are difficult to control, for example, if COVOD-19 patients also received other drug treatments when receiving TCZ or SAR treatment, and if the patient had other primary diseases that could affect the occurrence of DILI.

## Conclusion

IL-6 inhibitors are associated with the occurrence of AEs related to TCZ and SAR. We profiled DILI associated with the application of TCZ or SAR in COVID-19 patients with clinical characteristics, onset time and prognosis, based on the FAERS database. Clinicians should strictly monitor this potential DILI when treating COVID-19 patients with IL-6 receptor antagonists.

## Supplementary Information

**Additional file 1.** Information on COVID-19 patients treated with TCZ or SAR obtained from the FEARS database.

## Data Availability

The datasets used and/or analyzed during the current study are available from https://fis.fda.gov/extensions/FPD-QDE-FAERS/FPD-QDE-FAERS.html#collapse2
